# The Use of Wearable Systems for Assessing Work-Related Risks Related to the Musculoskeletal System—A Systematic Review

**DOI:** 10.3390/ijerph21121567

**Published:** 2024-11-26

**Authors:** Filippo Motta, Tiwana Varrecchia, Giorgia Chini, Alberto Ranavolo, Manuela Galli

**Affiliations:** 1 Dipartimento di Elettronica, Informazione e Bioingegneria (DEIB), Politecnico di Milano, Via Ponzio 34/5, 20133 Milan, Italy; manuela.galli@polimi.it; 2 Department of Occupational and Environmental Medicine, Epidemiology and Hygiene, INAIL, Monte Porzio Catone, 00078 Rome, Italy; t.varrecchia@inail.it (T.V.); g.chini@inail.it (G.C.); a.ranavolo@inail.it (A.R.)

**Keywords:** musculoskeletal disorders, ergonomics, review, wearable systems, work-related tasks

## Abstract

Work-related musculoskeletal disorders (WRMSDs) are a leading cause of chronic conditions among working-age adults. Preventing these disorders is crucial to reducing their impact, and quantitative analysis through sensors can help identify their causes and guide ergonomic solutions. This systematic review aims to compile research from 2000 to 2023 published in English and sourced from Web of Science, Scopus, or PubMed that examines workers’ movements during tasks using wearable sensor systems that are applicable in workplace settings. The goal is to identify the job sectors that have been studied and highlight tasks lacking ergonomic risk research. A total of 111 papers were selected through a screening process across three databases, assessed using the McMaster risk of bias tool. The studies span various job sectors and report on the use of different technologies for data collection and study population sizes. The review identifies existing research on WRMSD risks utilizing wearable systems in different job sectors, drawing attention to under-researched areas that warrant further study. It serves as a foundation for future research aimed at understanding the causes of WRMSDs and developing solutions supported by wearable technologies to mitigate these risks.

## 1. Introduction

Musculoskeletal disorders affect 1.71 billion people around the world, with lower back pain the leading cause of disability in 160 countries, source via WHO (https://www.who.int/news-room/fact-sheets/detail/musculoskeletal-conditions accessed on 6 November 2024). Among these disorders, the most common are work-related musculoskeletal disorders (WRMSDs), with more than 50% of workers declaring that they suffer from this issue (source via European Agency for Safety and Health at Work [1]). WRMSDs create problems of disability, lower quality of life, and absenteeism for the affected workers, which decreases the productivity of the job and increases healthcare costs. These disorders encompass a wide spectrum of conditions that affect the muscles, bones, tendons, ligaments, and other components of the musculoskeletal system, leading to discomfort, pain, and in severe cases, functional impairment [2].

As the global workforce continues to evolve, with related technological advancements and changes in work practices, the risk factors associated with WRMSDs have become more complex and nuanced. Given the large occurrence and severe consequences of these disorders, many attempts have been made over the past years to acquire information and apply different ergonomic strategies that can limit the occurrence of WRMSDs. Many studies have been performed using different systems and sensors to gather information about the causes of this widespread phenomenon [3,4,5]. In particular, the study of workers’ posture and motion can contribute to shedding light on the multi-factorial origin of the WRMSDs, as their causal factors include muscle strains and workload beyond the acceptable limits of the human musculoskeletal system, as well as cognitive and organizational elements. The posture and motion of workers can be acquired by different methods. The gold standard consists of optoelectronic systems [6] composed of cameras and markers attached to the body, usually paired with force platforms. In addition there are other methods based on wearable sensors [7]. Notably, these are applicable to the reality of the workplace, which cannot be said for optoelectronic systems or force platforms.

Understanding the intricate interplay between occupational demands, individual factors, and the ergonomic design of workspaces is paramount in developing effective strategies for prevention and intervention. By synthesizing current research findings, case studies, and best practices, this review seeks to contribute to the ongoing dialogue on WRMSDs, thereby fostering a deeper comprehension of the challenges at hand and encouraging the implementation of instrumental-based measures to create healthier and more sustainable work environments. This approach is aligned with the new European Committee for Standardization Workshop Agreement (CWA) 17938, titled “Guideline for introducing and implementing real-time instrumental-based tools for biomechanical risk assessment” [8]. As workplaces continue to evolve, addressing the complex issue of WRMSDs is essential for ensuring the well-being of the global workforce and optimizing overall economic productivity. The main objective of this study is to review all manuscripts that have monitored workers through wearable sensor networks in manual material handling (MMH) activities performed in the workplace. The collected documents are sorted by job sectors to provide an overall perspective on those tasks that have already been studied and on which jobs still need to be investigated. Furthermore, this review aims to investigate the main ergonomic indexes based on the kinematic, kinetic, and surface electromyographic signals that best characterize the physical effort of workers. This review could constitute a starting point for future research in physical ergonomics, which will need to be increasingly based on quantitative data from MMH activities performed directly in real occupational contexts. This document will be useful for researchers interested in carrying out a study on a certain work area and needing an overview of all previous studies.

## 2. Materials and Methods

This study was performed using the systematic review method proposed by the Preferred Reporting Items for Systematic Reviews and Meta-Analysis (PRISMA) [9].

### 2.1. Literature Search Strategy

Peer-reviewed journal publications were gathered from three of the biggest online databases: PubMed, Web of Science, and Scopus. The investigated papers were published between 2000 and August 2023 and were written in English. The three main interests of this review were human motion analysis, the different available technologies, and studies of work-related tasks; therefore, the following keywords grouped in different lists were used for searching the selected online databases:

- Human motion: “posture”, “movements”, “kinematics”;
- Acquisition methods: “Inertial measurement unit (IMU)”, “accelerometer”, “Surface electromyography (sEMG)”, “dynamometers”, “sensors”;
- Fields of interest: “ergonomics”, “manual lifting”, “pushing and pulling”, “handling of low load at high frequency”, “repetitive movements”.

Different combinations of keywords were used; in any combination, there were always at least six words: two from the “human motion” list, two from the “acquisition methods” list, and two from the “field of interest” list.

### 2.2. Screening Criteria

Among the many motion capture methods, this review reports only those studies based on systems that could be used in a real workplace scenario and not solely in a laboratory setting. For example, from the shortlisted papers, studies based on technologies such as marker-based optoelectronic systems and force platforms were found, but were excluded because it is not feasible to use these devices in the workplace due to their space requirements. Instead, studies based on IMU systems, sEMG, heart rate monitors (HRM), chest bands, and other wearable sensors were included. Moreover, in this review only those papers dealing with work-simulated tasks were considered, excluding those where the subjects performed standardized laboratory tasks. This decision was taken because the literature is lacking a systematic review with this focus, despite several existing literature reviews on methods that study work-related tasks [4,5]. However, these are either outdated or do not comprehend the same technologies. Therefore, all of the studies included in this review met the following criteria:

- Written in English;
- Based on a quantitative assessment;
- Based on data from wearable sensors;
- Based on data acquired during real or simulated work-related tasks;
- Focused on musculoskeletal disorders.

The exclusion criteria were as follows:

- Validity or feasibility studies;
- Studies about fall risk prevention, security in the workplace, sit–stand time, or vibration;
- Standardized laboratory tasks;
- Studies using force plates or optoelectronic system;
- Papers about the presentation of the study protocol.

**Figure 1:**
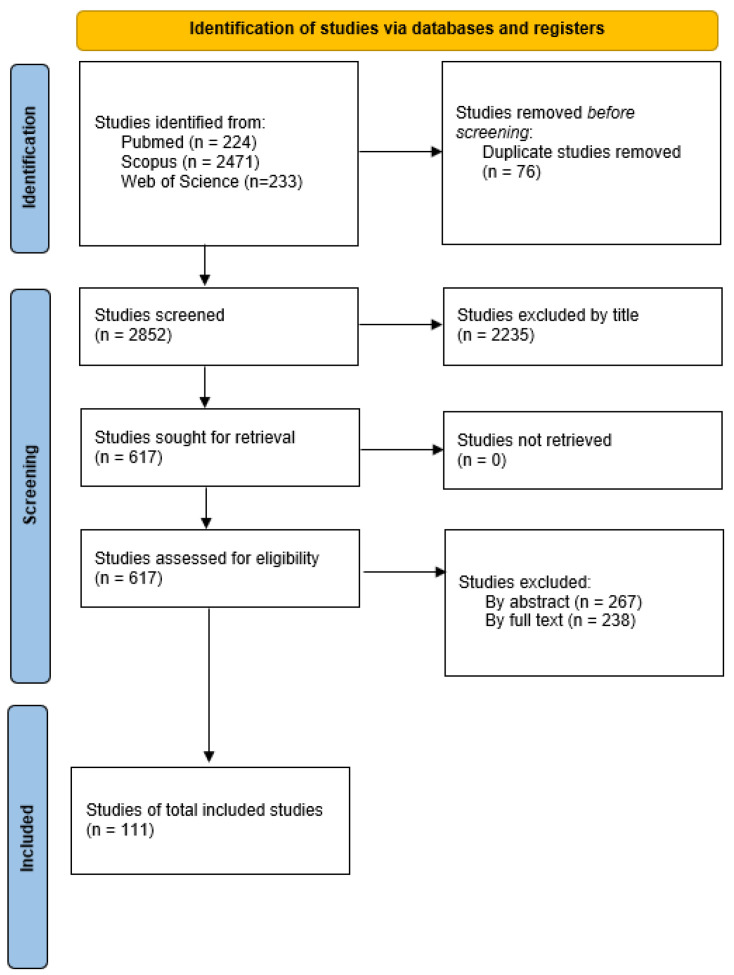
PRISMA flow diagram.

After gethering the papers, all duplicates were removed and all articles were filtered by the title following the inclusion and exclusion criteria. Then, the papers were screened by their abstracts and finally by their full texts based on the above-mentioned criteria. The results of these screening phases are depicted in Figure 1. The entire process was performed by the principal author; in doubtful cases, the entire team discussed whether or not the study should be included.

The selected articles were divided based on job sector to facilitate analysis and comprehension. The work described must have been carried out in accordance with The Code of Ethics of the World Medical Association (Declaration of Helsinki) for experiments involving humans, EC Directive 86/609/EEC for animal experiments, and the Uniform Requirements for manuscripts submitted to biomedical journals. The outcomes and variables for which data were sought consisted of the aim of the study, type of task executed, instrumentation used for data acquisition, investigated body parts, number of participants, ergonomics index of reference, and duration of the study. Non-described outcomes were reported as missing and not presented in the review.

### 2.3. Bias Risk Assessment

The McMaster Evidence Review and Synthesis tool for quality assessment of quantitative studies [10,11] is used to analyze the risk of bias of each included paper. Selection bias, study design flaws, managing of confounders and blinding, data collection methods, and the presence of dropouts were analyzed using this tool to assign a level of risk to each study. The results are reported in Table A1 in Appendix A. The studies that enrolled non-professional participants were still included, but this limitation was taken into account when evaluating the selection bias risk.

## 3. Results

Given the number of included papers, this review does not claim to present all the papers’ details; rather, it is intended to be used as a general overview. If a certain paper arouses interest, we invite the reader to use the literature reference to obtain more information. In presenting the different papers, only the quantitative methods are reported; therefore, questionnaires and clinical scales are not reported, as they are only semi-quantitative. The studies are divided into subsections based on the field of job as presented in Table 1: healthcare (Section 3.1 and Table 2), construction (Section 3.2), office work (Section 3.3), industrial (Section 3.4 and Table 3), and agro-food (Section 3.5 and Table 4). The studies that could not be assigned to any of these groups are described in Section 3.6 and Table 5. The studies on surgical or industrial tasks, with a sample size of less than ten subjects, are reported in Appendix A in Table A2 and Table A3, as their statistical power is lower compared to other studies on the same topics. Where present in the papers, the average duration of the data acquisition is reported between parentheses using the same unit of measurement as in the original paper: hours (h), minutes (min), or seconds (s). After the bias assessment, 92 papers were classified as weakly resistant to bias, while 19 were classified as moderately resistant. The complete results are reported in Table A1 in Appendix A.

**Figure 2:**
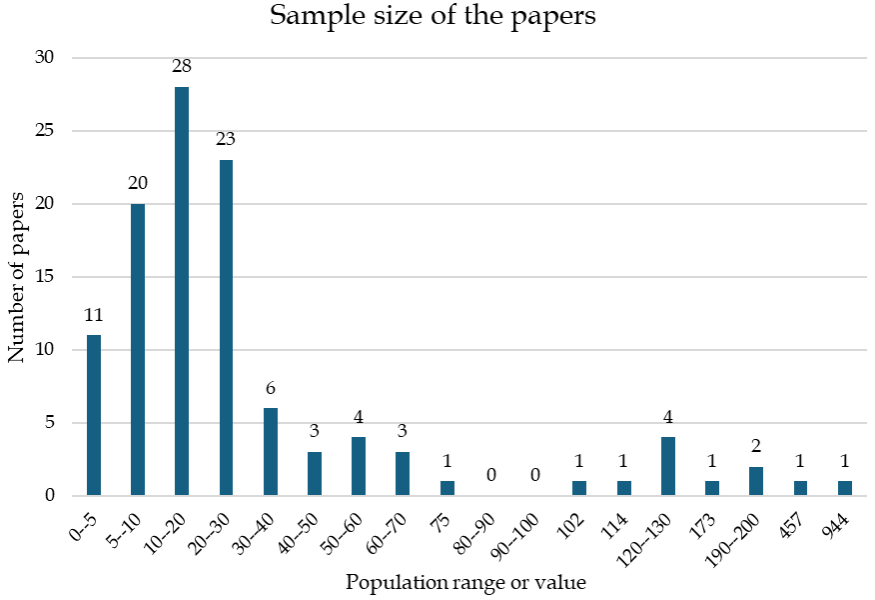
Number of papers for each range or value of sample size.

After reviewing the papers, a general overview was made. First, the distribution of the sample sizes of the different studies is presented in Figure 2. It can be observed that the large majority (59.2%) of the studies involved less than 30 subjects.

**Figure 3:**
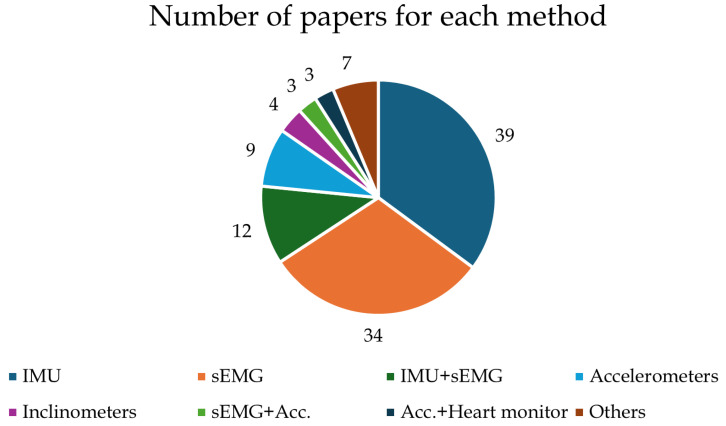
Number of papers for each motion capture system combination; “others” includes all combinations used in only one paper.

Figure 3 highlights the different wearable sensors used in the studies, indicating that IMUs and sEMG are the most used.

**Figure 4:**
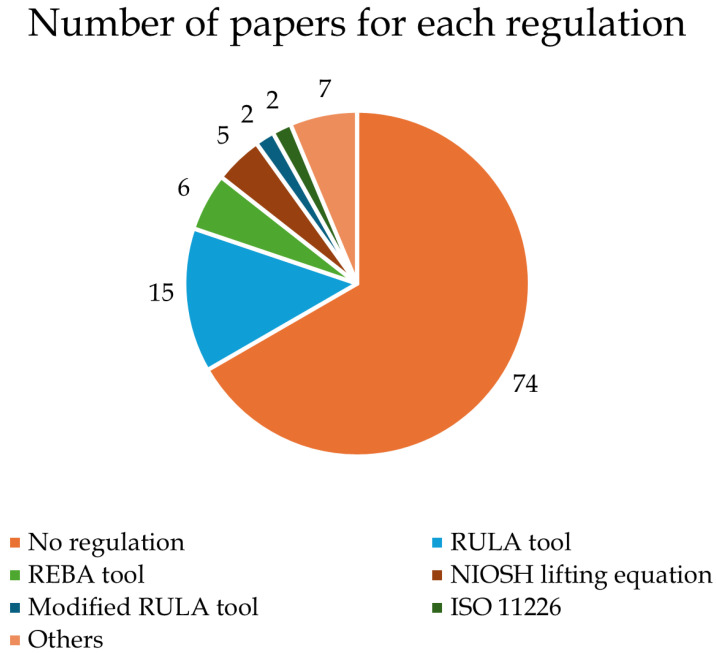
Number of papers for each ergonomic index; “others” includes all combinations used in only one paper.

Lastly, Figure 4 depicts the number of studies incorporating a regulation as a reference for comparing the results with ergonomic indexes. Only a small percentage of studies referred to a regulation; in most cases, this was the Rapid Upper Limb Assessment (RULA) tool [12], Rapid Entire Body Assessment (REBA) tool [13], or National Institute for Occupational Safety and Health (NIOSH) lifting equation [14].

### 3.1. Studies on the Healthcare System

This section contains all the studies about surgery, nursing, dentistry, and veterinary medicine. Starting with the sector of surgery, the first common objective is comparing laparoscopic, robotic, and open surgery types:

- Laparoscopic compared to robotic surgery: In [15], five IMUs were placed on the head, chest, waist, and biceps, while four sEMGs recorded the activity of trapezius and deltoid muscles of twenty surgeons and surgical trainees performing 29 robotic and 48 laparoscopic cases (average durations = 177 min and 112). From the results, muscular strain seems to be lower in robotic-assisted surgery. Other smaller studies of this comparison agree with this conclusion. In [16], one surgeon’s arms muscle activity was recorded by sEMG while performing thirteen laparoscopic and five robot-assisted operations. In [17], six surgeons with different experience levels were asked to perform basic surgery tasks with both laparoscopic and robotic platforms; their arm muscle activity recorded by sEMG showed that higher effort was borne during laparoscopic activity. In the last paper [18], five specialists performed four laparoscopic and four robotic-assisted surgeries while wearing six sEMGs on the trapezius and forearm muscles and four gravimetrical sensors on the neck, arms, and torso. The results agreed with those of the other studies on the lower muscular demand of robotic surgery.
- Laparoscopic compared to open surgery: In [19], sixteen vascular surgeons wore four IMUs on the upper arms, upper torso, and head to measure the time spent in dangerous postures during different surgical operations (average duration from 3.5 to 5.5 h). In another study [20] 24 surgeons performed 27 open and 22 laparoscopic surgeries (132 min) while wearing four IMUs on the shoulders, upper back, and head. Both studies found that open surgery causes more risky postures.
- Open compared to robotic surgery: In [21], a comparative study was conducted on 22 surgeons performing twelve open and ten robot-assisted surgeries (277 ± 84 min) while wearing two sEMGs on the trapezius and four IMUs on the head, neck, and upper arms. The results showed that robotic surgery incurs less muscular load. In a previous study [22], eight gynecologic surgeons performed both abdominal and robotic surgery (duration from 135 to 288 min) while wearing an accelerometer on the hip to measure the time spent at different levels of activity. In this study, no differences were found between the two approaches.

The second group of studies includes those that analyze impacts on ergonomics:

- New postures or methods: A few studies have analyzed the effects of different approaches to surgery. One study analyzed the difference between breast surgery procedures, where nipple-sparing vasectomy produced a higher workload compared to skin-sparing vasectomy as measured by four IMUs placed on the upper arms, upper back, and head of four different four surgeons [23]. Another study looked for differences between sitting and standing during vaginal hysterectomy and highlighted how the workload, measured based on the posture acquired with four IMUs placed on upper back, upper arms, and head, was worse on the trunk but better on the shoulders for the four seated surgeons compared to the nine standing surgeons [24]. In [25], the posture of four seated surgeons was measured by four IMUs on the chest, head, and upper arms during vaginal procedures (5–8 h) to compared the effects of using four different chairs, without significant differences in the comparison between chairs. In another study, [26] positive results were obtained in terms of muscle strain for a postural support chair during microscope work (average = 25 min) by ten clinicians wearing four sEMGs on neck and upper back.
- New tools: A number of studies have demonstrated the ergonomic effect of new handles for laparoscopy, sometimes without any ergonomic improvement. In [27], 57 subjects without any experience performed laparoscopic exercises (maximum duration 12 min) while wearing five sEMGs on the upper limbs and shoulders. In [28], eleven surgeons performed 40 laparoscopic procedures (28–29 min) using a rotatable handle while their muscle activity was recorded by six sEMGs on the trapezius and right arm muscles. Unlike the previous study, the device presented in [29] produced reduced muscle effort on the part of ten subjects with no surgical experience while performing four training exercises (maximum duration 20 min) while wearing six sEMGs on the muscles of both arms.

Lastly, there various studies [30,31,32,33,34,35,36,37] reported in Table 2 deal with different methods and sensors in a general analysis of the ergonomic risks of the job, analyzing muscle strain due to postures, method, duration, or equipment. All of these studies underline the risks that ergonomic concerns generate for surgeons.

Another group of papers investigated tasks involved in nursing. For example, in a study on upper body risks, [38] conducted measurements of 36 nurses by means of three IMUs placed on the upper arms and back, discovering that while there are rarely long periods of extreme posture over the course of a normal shift (8–12 h), there are very few opportunities for recovery. Another study of 27 nurses using one IMU placed on the hip found out that nurses with back injuries had lower lumbopelvic control compared to those without such injuries [39]. A study of eight nurses who wore an accelerometer for a full shift explored the relationship between back injuries and awkward trunk postures and explained how the two phenomena were related [40]. Lastly, a paper evaluating a new tool for reducing the lower back load during manual handling of patients showed how the device reduced the amount of time spent with the trunk flexed (as measured by one IMU on the sternum) for 28 caregivers [41].

In the dental health sector, the first of two studies on the use of magnification loupes did not find any quantitative difference in 25 dental hygienists’ posture based on measurements acquired by four accelerometers placed on the head and spine during a full-mouth exploration task (1.5 h) [42]. The other study found that the presented magnification loupe reduced trunk flexion for twelve dental hygienists who used it compared to twelve who did not, as measured by four IMUs placed on the head and spine during a mouth scaling task [43]. Finally, a study of 36 practicing dentists investigating different combinations of ergonomic supports to reduce the muscle activity of the dominant arm proved the ergonomic benefits of using magnification lenses or an ergonomic stool based on measurements taken with three sEMGs [44].

Finally, it is worth mentioning the only study concerning veterinarians, which aimed to characterize muscle activation via four sEMGs on the trapezius and deltoids as well as the posture of the neck and shoulders using five other IMUs during 26 live veterinary surgeries (average = 1.5 h) [45]. The study obtained results on the muscle activity of five veterinarians, showing similar effects to the activity of medical physicians.

Notably, these studies in the healthcare sector suffer from a strong risk of bias in the selection and blinding of participants, as they are aware of the study’s aims and are generally not representative of the entire population.

**Table 2:** Studies involving ergonomics assessments for surgical tasks.

First Author	Aim	Task	Subjects	Instrumentation	Body Segments
Asadi [30]	To propose a multi-modal approach for the live surgical work environment	laparoscopic surgery procedures (128 min)	12 surgeons	4 sEMG 4 IMU	upper back, upper and fore right an left arms, head
Norasi [31]	To quantify the postural demand, workload, and discomfort experienced by vascular surgeons and to identify the causal factors	vascular surgery (240 min)	16 surgeons	4 IMU	right and left upper arms, head and upper back
Yang [32]	To identify risk factors and assess intraoperative physical stressors, including the type of procedure and equipment used.	normal surgical shift (137 min)	53 surgeons	5 IMU	right and left upper arms, trunk, head
Yu [33]	To assess the ergonomics and workload for both assisting and console surgeons intraoperatively.	robotic prostatectomy (assisting = 142 min, performing = 129 min)	10 (console and assisting surgeons)	6 IMU	head, sternum, shoulders, pelvis
Smith [34]	To evaluate muscle fatigue and participant pain in the upper body muscles.	simulated laryngeal microsurgical tasks in 2 different postures (15 min each)	18 surgeons	7 sEMG	dominant side upper and forearm, deltoid, trapezius
Gold [35]	To compare initial ergonomic positioning between those who receive ergonomic teaching with those who did not.	microscopic temporal bone lab drilling (5 min)	14 otolaryngologists	3 IMU	head, sternum, lumbar region
Viriyasiripong [36]	To measure surgeons’ head movement during laparoscopic simulator.	tasks on a laparoscopic simulator (107–279 s)	19 medical students or surgeons	1 accelerometer	head
Khan [37]	To measure the impact of a structured training program in improving the ergonomic stress in trainee laparoscopic surgeons.	20 h of laparoscopic intra-corporeal suturing training	10 trainees, 3 experts	8 sEMG	right and left deltoids, upper and forearms

### 3.2. Studies on Construction

The construction industry is one of the most prominent sectors where WRMSDs are present; indeed, many more studies than those reported here are focused on this topic, as many studies investigating building-related tasks were excluded from this review for not meeting the inclusion criteria. The included studies can be divided into two main categories, namely, observational and interventional. Th observational studies focused on the following aspects:

- Muscle strain in lifting tasks: In [46], twenty student participants performed repetitive lifting tasks until fatigue occurred while their muscle activity was recorded using ten sEMGs placed on the arms, legs, and lower back. The results showed the highest muscle strain on the lumbar erector spinae.
- Effect of workers experience: One study [47] found no difference in ergonomic posture as measured by eight IMUs worn on the back, arms, and legs among six workers with different levels of experience performing 20 min of routine tasks. On the other hand, two studies [48,49] discovered that experts work in ergonomically safer ways. Both of these studies investigated 21 masonry workers with different levels of experience building a wall (25.5 min for the journeymen, 74 min for the novices) while wearing 17 IMUs to assess total body posture.

Despite their limitations, all of the above studies agree on the importance of quantitative assessment and sharing of knowledge with workers to reduce the risk related to their jobs, which can be very high due to the duties involved.

One of the interventional studies included in this review introduced three ergonomic workshops for 32 employees, with a control group of 48 workers [50]. The authors measured physical workloads at baseline using two IMUs on the upper back and thigh and four sEMGs on the trapezius and erector spinae for 3 months and 6 months, respectively. In the case group, the fatigue was reduced, but excessive workload events were not. Another interventional study [51] changed the work environment of thirteen masons divided into case and control groups by introducing a semiautomated lifter to help them build a wall. They found positive results in terms of reduced load on joints as measured by 17 IMUs placed on the entire body. The last interventional study [52] introduced a real-time system for ergonomic warnings based on two IMUs on the head and back, obtaining promising results for lifting tasks. It is worth mentioning that only one study focused on the risks for crane operators, in particular on the difference between 15 healthy operators and 17 operators with back or neck pain. The study found that the latter group used more awkward and dangerous postures over the course of an 8 h shift as measured by two inclinometers placed on the head and back [53].

Finally, two studies took into consideration 123 workers in both the healthcare and construction professions who wore two accelerometers for four consecutive days. The studies focused on standing posture [54] and forward bending [55], but found no clear association with pain.

The major risk of bias for the studies on construction is related to the blinding method, as the subjects are aware of being assessed and know the study’s aim.

### 3.3. Studies on Office Work

All the studies in this category are focused on the ergonomics of sitting. One study focused on finding the best torso angle for twelve volunteers, starting from data collected by three IMUs placed on the spine [56]. Another study analyzed the activity of the trapezius muscles (as recorded by two sEMG) and how it related to the arm posture (as measured by two inclinometers on the arm and leg) of 26 computer workers [57], and did not find any stressful factors. Another study focused on the difference in motor control between ten subjects with chronic pain and thirteen healthy control subjects via two sEMGs placed on the trapezius, finding that during five computer tasks of 5 min each, the pathological cases had a higher motor unit potential rate for that muscle [58]. Two cases analyzed an ergonomic intervention for the sitting posture. In the first [59], fourteen workers used two different chairs for 5–6 h while an sEMG recorded their upper back and neck muscles. The other study [60] used two sEMGs to measure the trapezius muscle activity of 67 workers during typing tasks of 5 min on four different workstation designs. Another study involving six workers [61] added a biofeedback system based on an accelerometer placed on the neck to monitor posture during 5 h of work. In all of the above cases, the interventions successfully reduced the load on the muscles. Other studies focused on workstation equipment, such as different chairs. In [62], the authors found no significant difference in activation of trapezius and erector spinae as recorded by four sEMGs for twelve volunteers performing 100 min of standardized tasks. The difference between a home office and a real office [63], with the office workstation showing a more ergonomic setup for twenty subjects recorded over 20 min by a full-body IMU system consisting of 17 IMUs. Again, the major risk of bias in these studies is their lack of blinding methods.

### 3.4. Studies on Industrial Tasks

The studies included in this section vary for the specific field, work tasks executed, and technologies used for assessment; however, they all involve the industrial sector. Only three studies [64,65,66] used an ergonomic index as reference, all of which used the Rapid Upper Limbs Assessment (RULA) tool. All papers are reported in Table 3 along with their main information [67,68,69,70,71,72,73,74,75,76,77,78,79,80,81,82,83,84,85,86].

The risk of bias is related to the blinding method for the majority of the studies, with also a large percentage of studies with risks related to the participants’ selection.

**Table 3:** Studies from the industrial sector.

First Author	Aim	Task	Subjects	Instrumentation	Body Segments
Villalobos [66]	Present an application of IMUs to measure human activity, and the use of AI to perform task classification and ergonomic assessments in workplace settings.	normal work in a slaughterhouse during a morning shift (8 h)	20 meat cutters	1 IMU	dominant wrist
Karakikes [67]	To develop a wearable wrist-to-forearm angle measurement system.	screwing task for two screwdrivers (one long and one short)	12 volunteers	3 IMU	dominant upper and forearm
Tian [68]	To assess the postures that were commonly used in automobile chassis repair operations, and to evaluate shoulder girdle muscle fatigue.	maintaining 4 different postures with different dumbbells for 60 s.	15 students	2 sEMG	right trapezius and deltoid
Zare [69]	To assess the proportion of time in risky postures for the main joints of the upper limbs in a truck assembly plant and explored the association with musculoskeletal symptoms	task of workstations of a truck assembly plant (2 h)	13 workers	3 accelerometers, 2 inclinometers and 2 electro-goniometries	right and left upper and forearms, back, neck and hands
Michaud [70]	To describe an ergonomic intervention to reduce lateral epicondylitis in the workstation of a textile logistics centre	pick up, carry and throw some items onto the carousel (2 h).	93 workers first phase—27 s phase	7 IMU	trunk, right and left upper and forearms, hands
Reinvee [71]	To evaluate the ergonomic benefits of an angle grinder with a rotatable main handle in a cutting task.	use an angle grinder to cut a horizontal steel rod using three wrist postures	11 workers	3 sEMG and force-sensing-resistor-based force glove	Dominant upper an forearm
Bergsten [72]	To study the extent to which shoulder pain developed during single work shifts of flight baggage handlers.	work shift of handling flight baggage (2.7 h)	44 baggage handlers	2 accelerometers	upper arms
Palm [73]	To assess potentially harmful work exposure of arm elevation, by comparing work time and leisure, in a population with diverse work tasks.	diverse work tasks, for 1–4 days	197 workers	4 accelerometer	right upper arm, back, hip
Moriguchi [74]	To evaluate posture, forces required and perceived exertion when loading and unloading the ladder on a utility truck.	loading/unloading a ladder on vehicles	9 overhead line workers	2 inclinometers, dynamometer	shoulders
Conforti [75]	To examine the motion during lifting and repositioning of different loads.	lifting and repositioning (4 s) performed in two conditions, safe and unsafe	10 workers	8 IMU	total body
Ershad [76]	To investigate the response of trunk muscles in subjects with chronic non-specific low back pain (CNLBP) while holding unstable dynamic loads.	12 tasks of static and dynamic holding of loads in neutral positions for 5 s	12 males with CNLBP and 12 controls	6 sEMG	right side trunk
Johansen [77]	To investigate gender difference in the coordination of the subdivisions of the trapezius muscle during a repetitive box-folding movement task.	repetitive box-folding task for 34 min	11 males and 11 females volunteers	3 sEMG	dominant trapezius
Jakobsen [78]	To investigate the influence of sex, age, muscle strength, and cardiovascular fitness on manual lifting patterns among blue-collar workers.	manual lifting (5–10 s)	173 employees (14 workplaces)	6 sEMG and 2 accelerometers	dominant thigh, shoulder and low-back
Porta [79]	To investigate age-related differences in patterns of trunk flexion of workers in the metal working industry.	normal work shift in the metal working industry (8 h)	33 workers	1 IMU	trunk
Poosanthanasarn [80]	To assess the causes of injuries in sections of a factory, and to improve working conditions using an ergonomics intervention program (EIP)	work shift in pressing and storage sections of the metal auto parts factory	35 (with EIP) and 17 (no EIP)	4 sEMG (5 min, 3 times in a day)	back
Tjøsvoll [81]	To assess the physical work demands of onshore petroleum maintenance workers.	onshore petroleum maintenance workers day (recorded up to 6 days, 24 h each)	46 maintenance workers	5 accelerometers and a heart rate sensor	dominant upper arm, upper and lower leg, trunk
Wahlström [82]	To assess upper body postural exposure among airport baggage handlers and determine whether exposure differs between workers at the ramp and baggage sorting areas.	full work shift (8 h) at the airport of baggage handling	27 baggage handlers	2 inclinometers	trunk and dominant shoulder
Skovlund [83]	To measure muscular workload during stocking activities and the thirteen most common work tasks across supermarket chains	transport, stocking and lifting (10–20 min)	75 supermarket workers	6 sEMG	back and shoulders
Sander De Bock [84]	To assess the effect of an exoskeleton, Exo4Work, on muscles during simulated occupational work.	different overhead and non-overhead tasks (20 min)	22 healthy volunteers	7 sEMG	trunk and right and left upper and forearms
Gupta [85]	To investigate the dose–response relation between device-measured forward bending at work and prospective register-based risk of long-term sickness absence (LTSA).	normal work shift (457 min)	944 workers (93% blue-collar jobs)	3 IMU	upper back, dominant upper arm and thigh
Villumsen [86]	To investigate the association between forward bending of the trunk and low back pain intensity among workers and whether the level of social support modifies the association.	normal workday and leisure day (19.6 h and 22.7 h)	457 blue-collar workers	2 accelerometers	trunk

### 3.5. Studies on Agro-Food Sector Tasks

In this last section, all the studies about the agro-food sector are described and summarized in Table 4. They analyse different tasks such as: harvesting and fruit picking [87,88,89,90,91], cow milking [92,93] or routine activities [94].

For the majority of these studies, the main risk of bias is related to the blinding and participant selection methods.

**Table 4:** All the studies on agri-food sector tasks.

First Author	Aim	Task	Subjects	Instrumentation	Body Segments
Chan [87]	To create and evaluate an upper extremity musculoskeletal model of the oil palm harvesting motion and to assess the associated risk.	harvesting of oil palm (1 min)	6 harvesters	5 sEMG and 6 IMU	back, right and left upper and forearms
Teo [88]	Quantification of muscles activations and joints range of motions during oil palm fresh fruit bunch harvesting and loose fruit collection	fresh fruit bunch harvesting and loose fruit collection (3 min each)	8 harvesters and 8 collectors	6 IMUs and 7 sEMG	right and left upper and forearms and trunk
Thamsuwan [89]	To present an approach to characterize the repetitive motions of the upper arms based on direct measurement using accelerometers	picking apples from trees (30 min)	24 harvester	2 accelerometers	upper arms
Roquelaure [90]	To evaluate biomechanical strains on the hand–wrist system during grapevine pruning.	using the hand-powered pruning shears during grapevine pruning	6 vineyard workers	1 sEMG and 1 electrogoniometer	right hand and wrist
Komarnicki [91]	To present the relationship between the ergonomics in the work of a strawberry picker and quality of picked fruit	strawberries picking (10 min)	1 picker	4 sEMG	low back, dominant hand
Masci [92]	To compare the upper limb muscle activity during milking tasks between workers at large-herd U.S. dairies and small-herd Italian dairies.	cow milking (1 h)	65 dairy workers	5 sEMG	dominant upper and forearms
Mixco [93]	To quantify upper limb muscle activity among workers performing milking tasks in large-herd dairy parlors.	cow milking (45–90 min)	26 dairy workers	5 sEMG	dominant upper and forearms
Fethke [94]	To quantify and compare exposure to biomechanical factors among farmers performing a variety of routine agricultural activities.	variety of routine agricultural activities (4 h)	55 farmers	5 sEMG and 5 IMU	trunk, dominant upper arm and wrist

### 3.6. Studies on Other Work Tasks

In this last section, all the studies that could not be assigned to any other group are described and summarized in Table 5. The investigated job sectors are mail sorting [95,96,97], cleaning [98,99,100,101], gardening [102,103,104], hairdressing [105], policing [106,107], caregiving [108,109,110], and piloting [111].

**Table 5:** Studies with no other categorization.

First Author	Aim	Task	Subjects	Instrumentation	Body Segments
Lind [95]	To present a new sensor-based system for preventive measures of risk assessments.	mail sorting (repetitive task)	16 novice	2 IMU	right and left upper arms
Silva [96]	To evaluate postal workers’ pain symptoms, movements and proposing preventive measures.	parcel processing activity for delivery	32 workers	17 IMU	total body
Hemphäläa [97]	To assess the benefits of visual ergonomics intervention in mail sorting facilities	mail sorting	from 12 to 27 subjects	2 sEMG, 2 inclinometers	right and left trapezius, upper back and head
Madeleine [98]	To investigate the relation between self-reported pain, muscular activity and postural load during cleaning tasks.	usual cleaning tasks in a laboratory and a lecture room	18 cleaners	4 sEMG, 1 accelerometer	right and left trapezius, erector spinae and back
Unge [99]	To clarify if differences in the physical workload, the psychosocial factors and musculoskeletal disorders can be attributed to work organizational factors.	normal work shift (8 h)	24 hospital cleaners (traditional work organization) and 22 (extended one)	2 sEMG and 5 accelerometers, 2 electrogoniometers	trapezius, head, upper back, right upper arm and wrist
Lee [100]	To investigate whether work pace is a critical indicator for predicting a janitor’s risk of work-related musculoskeletal disorders	different cleaning tasks (11.7–74.2 min)	13 janitors	HR monitor, pedometer, 1 accelerometer	chest and trunk
Kiermayer [101]	To quantitatively determine the musculoskeletal load of washroom employees in an animal facility with a holding capacity of 35,000 rodent cages	cleaning and managing of rodent cages (335 min)	2 workers	CUELA system [112]	trunk and upper and forearms
Yang [102]	To measure the activity of the operator when using the bush cutter for different landscape tasks.	using 2 types of brush cutters in the 3 working conditions (30 min each)	6 workers	10 sEMG	trunk
Yang [103]	To demonstrate which working condition causes the most muscle fatigue, evaluate work fatigue accurately, and reduce WRMSDs in garden workers	hedge pruning (60min)	120 gardeners	8 sEMG and 17 IMU	total body
Landekic [104]	To investigate the impact of three different chainsaw starting methods on the postural load of the worker and its association with personal and occupational factors.	starting a chainsaw	28 workers	17 IMU	total body
Wahlström [105]	To describe female hairdressers’ movement, including the variability between hairdressers, between days within hairdresser, and between tasks.	customer tasks and auxiliary non-customer tasks, including breaks (4 days of work)	28 female hairdressers	2 inclinometers	deltoids
Vera-Jiménez [106]	To analyse Biomechanical Parameters in Police Physical Intervention Techniques for Occupational Risk Prevention.	control of an opponent by a police officer (5 s)	1 female officer	19 IMU	total body
Mohammad [107]	To evaluate the effect of lumbar support with a built-in massager system on spinal angle profiles among traffic police riders	riding the high-powered motorcycle (20 min)	24 police riders	1 smart shirt (5 inclinometers)	back
Wong KC [108]	To determine the time spent in different static trunk postures during a typical working day of workers in a special school for severe handicaps.	typical working day of caring children with severe handicaps (6 h)	18 workers (low back) pain and 15 healthy subjects	1 accelerometer	back
Holtermann [109]	To assess the physical work demands with accelerometers and workplace observations of childcare workers.	normal work week for childcare workers (34.9 h)	199 childcare workers	5 accelerometers	trunk, dominant upper arm, calves and right thigh
Tjøsvoll [110]	To assess physical work demands in home care, using wearable sensors.	home caring (24 h for 6 days)	114 home workers	5 accelerometers and heart rate sensor	dominant upper and lower leg, upper back and deltoid
Balasubramanian [111]	To investigate the effects of a helicopter flight on pilots’ back and shoulder muscles.	pre and post flight (60 s)	8 Coast Guard helicopter pilots	4 sEMG	bilateral trapezius, erector spinae

For the majority of these studies, the risk of bias is related to the blinding and participant selection methods, as the subjects were almost always aware of the study aims and purpose of the research.

## 4. Discussion

This systematic review evaluates the use of wearable systems applicable in real work scenarios for analyzing the ergonomic risks of workers while performing job tasks. To reach this aim, we found 111 eligible articles, in the main text from [15,16,17,18,19,20,21,22,23,24,25,26,27,28,29,30,31,32,33,34,35,36,37,38,39,40,41,42,43,44,45,46,47,48,49,50,51,52,53,54,55,56,57,58,59,60,61,62,63,64,65,66,67,68,69,70,71,72,73,74,75,76,77,78,79,80,81,82,83,84,85,86,87,88,89,90,91,92,93,94,95,96,97,98,99,100,101,102,103,104,105,106,107,108,109,110,111] as well as others in Appendix A). During the process of screening the papers, many studies were excluded due to the use of technologies that are inappropriate for real work scenarios, such as optoelectronic systems or force plates. Although these methods are the most accurate and represent the gold standard in motion analysis, their application in real work fields is impracticable. Most of the ergonomic analyses included in this review observed that the investigated jobs could lead to WRMSDs, as they have features that represent risks of poor postures or increased activation of muscles. In detail, the included studies evaluated different work activities using an instrument-based approach. The measurement results show that most tasks in the industrial sector usually have poor ergonomic conditions that could lead to the onset of low back pain [69,70,73,75,78,80,83]. It is known in fact that lower back pain conditions are particularly common WRMSDs among occupational groups that involve heavy lifting or prolonged sitting, such as industrial workers and nurses [113], as reported in several studies included in the present review [38,39,40,41]. Perhaps because the industrial sector and healthcare sectors represent some of the most high-risk work areas, 61% of the reviewed studies concern these two sectors (43 from healthcare and 25 from the industrial sector; see Table 1). Among the other studies included in this review, some studies in the office work [58,59,60,61,62,63] and other [97,98,99,111] categories reported head posture and upper back muscle monitoring as key elements in the reduction of ergonomic risk. Indeed, neck pain is another frequently reported WRMSD in settings where prolonged computer use or repetitive tasks are common, i.e., office work. Research has shown that neck pain affects approximately 39.3% of workers in various industries [114]. This condition is often linked to poor ergonomic practices and prolonged static postures, which can exacerbate muscle strain and discomfort [115]. Poor ergonomic wrist conditions were also reported in most of the included studies on surgery tasks [16,17,18,28,29,30,34,37] and material handling [66,67,69,71,84,87,88,90,92,93,94,99,101], possibly explaining the cause of this common condition. Indeed, upper extremity disorders, particularly those affecting the wrists, are highly prevalent. Studies among garment workers and other manual laborers indicate that wrist pain is a common complaint, with reported rates as high as 67% in certain populations [116]. Most interventional studies reported how risk exposure was decreased thanks to the studied changes, underlining the potential of tailored interventions [23,24,25,26,27,28,29,35,37,41,43,44,50,51,52,60,61,67,70,71,80,84,97,107]. This brief overview of the most common WRMSDs highlights that most work tasks can lead to the occurrence of such disorders. However, as reported in Table 1, aside from the healthcare sector, where WRMSDs are relatively well-documented from [15,16,17,18,19,20,21,22,23,24,25,26,27,28,29,30,31,32,33,34,35,36,37,38,39,40,41,42,43,44,45], other sectors remain underexplored or even entirely unstudied despite the results studies pointing to problems related to the ergonomic conditions of workers in those sectors. Recent advancements in wearable technology could help to fill these gaps by offering promising tools for field-based risk assessment. Figure 3 shows that among the wearable sensors used in the included studies, inertial measurement units (IMUs) and surface electromyography devices (sEMGs) were the most used (73% of the studies). The reason for this is their ease of use and the importance of the data they provide. In fact, it is possible to use IMUs to measure kinematic variables (i.e., angles joints), and consequently subjects’ posture as well, which can then be used in ergonomic indices such as RULA and REBA. On the other hand, sEMG devices provide data on muscle activity, which can be used to compare the level of muscle engagement under different conditions to assess which are less fatiguing [15,16,17,18,21,26,27,28,29,34,37,44,50,59,60,71,80,84,92,93,97,99,103]. Both systems are usually transportable, and as such can be used in the field and not just in the laboratory. Their use in the field allows researchers to assess the actual risk to workers in real-time [117] and without the need for laboratory conditions that could affect the veracity of the results. Indeed, without standards that can guide researchers, it is not possible to transform the analysis of subjects’ efforts into a proper risk analysis. This need has already been discussed in the literature [118], and is partially assessed by the new CWA 17938 [8]; however, more work needs to be done in order to create new guidelines for quantitative work risk assessment based on quantitative measurement methods. Notably, the results of this review (see Figure 4) reveal that only 37 of the reviewed studies compared their results against a formalized ergonomic risk standard. The most widely used are RULA and REBA, which are based on the joint angles, exchanged forces, support, symmetry, and repetitiveness. The NIOSH lifting equation is also used; it is based on the geometry of the lifting task, its frequency, symmetry, and the amount of weight lifted. None of these directly consider the muscle strain and activity required by the worker; therefore, most studies based on sEMG do not report a reference index. Kim et al. [119] proposed a multi-index approach that utilizes sEMG to assess the physical load on workers during typical manufacturing tasks. Their findings indicate that specific ergonomic indexes can effectively correlate with muscle load, providing a comprehensive framework for evaluating workplace ergonomics. This study underscores the importance of considering individual worker characteristics when assessing ergonomic risks. Following this idea, an update to the most common ergonomic indexes including muscle activity assessment and worker characteristics could lead to more effective safety monitoring and greater use of these regulations in ergonomics studies. In recent years, the scientific literature has suggested that emerging technologies such as markerless motion capture systems and AI-based algorithms [120,121] may hold the potential to revolutionize ergonomic risk assessment thanks to their non-intrusive nature and ability to capture movement data without restricting workers’ range of motion. However, their application in real-world work scenarios remains limited at present, suggesting an opportunity for future research to explore and validate these tools in diverse occupational settings. The studies selected in this review have several limitations. In detail, as illustrated in Figure 2, 53% of the reviewed studies involved fewer than twenty participants, with some studies including even fewer than ten. This small sample size, often composed of convenience samples rather than randomized groups [34,39,41,42,43,70,79,94,100,107,109,110,122,123,124,125,126], limits the generalizability of findings and the strength of the conclusions that can be drawn. Additionally, many studies took place in laboratory settings where tasks were simulated [26,27,29,34,35,43,63,68,75,122,123,127,128] rather than analyzed in the real work environment. While these studies provide valuable insights, their results may lack external validity, as they do not account for the dynamic and varied conditions of actual workplaces. In addition, this review has several limitations that should be acknowledged. Only three databases were used to source the studies, potentially excluding relevant articles from other sources. Additionally, the screening process was conducted primarily by a single author, which may have introduced bias. Finally, due to methodological differences across the included studies, direct comparison of their results was not feasible, limiting the ability to synthesize findings into definitive conclusions.

## 5. Conclusions

This systematic review has underlined how quantitative instrumental measurements of workers provide a more accurate representation of exposure levels and safety monitoring, allowing for the customization of ergonomic interventions. This review can represent a starting point for future research focusing on understanding the causes of WRMSDs and creating solutions to mitigate them. In order to capture the real risk exposure, future studies should create new ways to assess ergonomic risks based on quantitative data that can be used in the actual workplace rather than solely in the laboratory. Another research direction could be the improvement of ergonomic indexes, including methods for quantitative measurement and consideration of workers’ different characteristics. A holistic approach with the collaboration of different professionals should be taken into consideration in order to analyze workers on a more general level rather than only in terms of their kinematics. To conclude, suggested improvements in risk assessment methods could contribute to an overall reduction in WRMSDs and improve occupational wellbeing in a variety of sectors.

## Figures and Tables

**Table 1:** Number of papers per subsection (*two studies with subjects from both healthcare and construction are reported at the end of Section 3.2).

Healthcare System	Construction	Office	Industrial	Agro-Food	Others
43	8(*+2)	8	25	8	17

## Data Availability

No new data were created.

## Abbreviations

The following abbreviations are used in this manuscript:

WHO	World Health Organization
WRMSDs	Work-Related Musculoskeletal Disorders
PRISMA	Preferred Reporting Items for Systematic Reviews and Meta-Analysis
IMU	Inertial Measurement Unit
sEMG	Surface Electromyography
sEMGs	Surface Electromyography Devices
HRM	Heart Rate Monitor
RULA	Rapid Upper Limb Assessment
REBA	Rapid Entire Body Assessment
NIOSH	National Institute for Occupational Safety and Health

## Appendix A

**Table A1:** Resistance to bias assessment results for all the studies; W = weak resistance, M = moderate resistance, S = strong resistance, NA = not applicable.

PAPER	Selection Bias	Study Design	Confounders	Blinding	Data Collection Methods	Drop-Outs	TOTAL
Monfared [15]	W	M	S	W	S	S	W
Zihni [16]	W	NA	NA	W	S	NA	W
Zihni [17]	W	NA	NA	W	S	NA	W
Kramer [18]	W	NA	NA	M	S	NA	W
Davila [19]	W	NA	NA	W	S	NA	W
Yang [20]	W	NA	NA	W	S	NA	W
Fan [21]	W	NA	NA	W	S	NA	W
Collins [22]	W	NA	NA	W	S	NA	W
Hallbeck [23]	W	NA	NA	M	S	NA	W
Singh [24]	W	M	W	S	S	S	W
Singh [25]	W	S	W	S	S	S	W
Vijendren [26]	W	M	W	W	S	S	W
Steinhilber [27]	M	M	NA	W	S	NA	M
Kraemer [28]	W	M	NA	W	S	NA	W
Gonzalez [29]	W	M	NA	W	S	NA	W
Asadi [30]	W	NA	NA	W	S	NA	W
Norasi [31]	W	NA	NA	W	S	NA	W
Yang [32]	M	NA	NA	W	S	NA	M
Yu [33]	W	NA	NA	W	S	S	W
Smith [34]	W	S	S	W	S	S	W
Gold [35]	W	S	S	W	S	S	W
Viriyasiripong [36]	W	NA	NA	W	S	NA	W
Khan [37]	W	NA	NA	W	S	NA	W
Schall [38]	W	NA	NA	S	S	NA	M
Babiolakis [39]	W	M	W	W	S	S	W
Nourollahi [40]	M	NA	NA	W	S	NA	M
Omura [41]	S	S	S	W	S	S	M
Ludwig [42]	W	S	E	E	S	S	W
Ludwig [43]	W	S	W	W	S	S	W
Garcia [44]	W	NA	NA	W	S	NA	W
Asadi [45]	W	NA	NA	W	S	NA	W
Antwi [46]	W	NA	NA	W	S	NA	W
Valero [47]	W	NA	NA	W	S	NA	W
Alwasel [48]	W	M	NA	W	S	NA	W
Ryu [49]	M	M	NA	W	S	NA	M
Brandt [50]	M	S	W	M	S	M	M
Ryu [51]	W	S	NA	W	S	NA	W
Yan [52]	W	W	W	W	S	S	W
Nourollahi [53]	W	W	W	W	S	S	W
Lunde [54]	M	NA	NA	W	S	NA	M
Lunde [55]	M	NA	NA	W	S	S	M
Estrada [56]	W	NA	NA	W	S	NA	W
Mork [57]	W	NA	NA	W	S	NA	W
Kallenberg [58]	W	M	W	W	S	S	W
Lasanen [59]	W	M	NA	W	S	S	W
Dowler [60]	M	M	NA	W	S	S	M
Breen [61]	W	M	NA	W	S	S	W
Ellegast [62]	W	M	NA	W	S	S	W
Holzgreve [63]	W	M	NA	W	S	S	W
Villalobos [66]	W	NA	NA	W	S	NA	W
Karakikes [67]	W	M	NA	W	M	S	W
Tian [68]	W	M	NA	W	S	S	W
Zare [69]	W	NA	NA	W	S	NA	W
Michaud [70]	M	M	NA	W	S	S	M
Reinvee [71]	W	M	NA	W	S	S	W
Bergsten [72]	M	NA	NA	W	S	NA	M
Palm [73]	S	NA	NA	W	S	NA	M
Moriguchi [74]	W	NA	NA	W	S	NA	W
Conforti [75]	W	M	NA	W	S	S	W
Cite [76]	W	M	NA	W	S	S	W
Johansen [77]	W	NA	NA	W	S	NA	W
Jakobsen [78]	S	NA	NA	W	S	NA	M
Porta [79]	W	NA	NA	W	S	NA	W
Poosanthanasarn [80]	W	W	W	W	S	S	W
Tjøsvoll [81]	W	NA	NA	W	S	NA	W
Wahlstrom [82]	W	NA	NA	W	S	S	W
Skovlund [83]	M	NA	NA	W	S	NA	M
De Bock [84]	W	M	NA	W	S	S	W
Gupta [85]	S	NA	NA	W	S	S	M
Villumsen [86]	S	M	S	W	S	S	M
Chan [87]	W	NA	NA	W	S	NA	W
Teo [88]	W	NA	NA	W	S	NA	W
Thamsuwan [89]	W	NA	NA	W	S	NA	W
Roquelaure [90]	W	NA	NA	W	S	NA	W
Komarnicki [91]	W	NA	NA	W	S	NA	W
Masci [92]	M	M	NA	W	S	NA	W
Mixco [93]	W	NA	NA	W	S	NA	W
Fethke [94]	M	NA	NA	W	S	NA	W
Lind [95]	W	M	NA	W	S	NA	W
Silva [96]	W	NA	NA	W	S	NA	W
Hemphala [97]	W	M	NA	W	S	M	W
Madeleine [98]	W	NA	NA	W	S	NA	W
Unge [99]	M	S	W	W	S	NA	W
Lee [100]	W	NA	NA	W	S	NA	W
Kiermayer [101]	W	NA	NA	W	S	NA	W
Yang [102]	W	M	NA	W	S	S	W
Yang [103]	M	M	NA	W	S	S	M
Landekic [104]	W	NA	NA	W	S	NA	W
Wahlström [105]	W	NA	NA	W	S	NA	W
Mohammad [107]	W	S	W	W	S	S	W
Wong KC [108]	W	M	NA	W	S	NA	W
Holtermann [109]	W	NA	NA	W	S	S	W
Tjøsvoll [110]	S	NA	NA	W	S	S	M
Balasubramanian [111]	W	NA	NA	W	S	S	W
END OF PAPERS IN THE MAIN TEXT							
Vignais [64]	W	NA	NA	W	S	NA	W
Colim [65]	W	M	NA	W	S	NA	W
Baird [122]	W	M	NA	W	S	NA	W
Wright [123]	W	NA	NA	W	S	NA	W
Reddy [124]	W	M	NA	W	S	NA	W
Katsarou [125]	W	M	NA	W	S	S	W
Kersten [126]	W	M	NA	W	S	S	W
Zhang [127]	W	NA	NA	W	S	NA	W
Uhrich [128]	W	NA	NA	W	S	NA	W
Arrighi [129]	W	M	NA	W	S	NA	W
Carbonaro [130]	W	NA	NA	W	S	NA	W
Ferrari [131]	W	NA	NA	W	S	NA	W
Kramp [132]	W	NA	NA	W	S	NA	W
Wang [133]	W	NA	NA	W	S	NA	W
Brents [134]	W	NA	NA	W	S	NA	W

**Table A2:** All studies, including those with a lower population.

First Author	Aim	Task	Subjects	Instrumentation	Body Segments
Baird [122]	To measure the effects of posture during ergonomically good and bad positions during laryngoscopy	laryngoscopy assumed four ergonomically distinct standing positions (4 min)	8 surgeons	12 sEMG	upper and forearms, shoulders, back and thighs
Wright [123]	To assess the effects of different surgeon positions and ureteroscope types on muscle activation	simulated flexible ureteroscopy tasks	3 surgeons	7 sEMG	dominant upper and forearms
Reddy [124]	To stratify ergonomic risk in a urologic microsurgeon using the 4K-3D exoscope versus the operating microscope.	vasovasostomy, vasoepididymostomy, varicocelectomy, testicular sperm extraction, and micro epididymal sperm aspiration	1 urologist	3 IMUs	head and upper arms
Katsarou [125]	To evaluate the effectiveness of a weightless exoskeleton-based radiation protective ensemble, in reducing ergonomic posture risks.	endovascular procedures while wearing a traditional LA (34.66 min) or the StemRad MD radiation protection system (48.4 min)	9 interventionists	1 IMU	upper back
Zhang [127]	To evaluate mental and physical workload of laparoscopic surgeons	Complete a laparoscopic surgery using a simulator.	4 surgeons and 10 predoctoral students	eye-tracking, 8 sEMG	eyes, upper and forearms
Uhrich [128]	To assess the level of muscle activity and compare the effects of fatigue, monitor placement, and surgical experience.	4 tasks of simulated skills for laparoscopy (2 min)	4 attending and 4 resident surgeons	6 sEMG	back, shoulders, upper legs
Arrighi [129]	To objectively evaluate the ergonomic positions of trainee and attending surgeons	functional endoscopic sinus surgery (54.80 attendings, 63.45 min trainees).	6 surgeons (two attendings and four trainees)	11 IMU	total body
Carbonaro [130]	To present a sensor-based platform specifically aimed at monitoring the posture during actual surgical operations	laparoscopic anterior resection of the rectum (3 h)	1 surgeon	3 IMUs	spine and neck
Ferrari [131]	To compare the effect of repeated manual and automated impactions on the user’s muscle	repetitive mallet swings to impact a surgical handle	7 surgeons	8 sEMG	dominant upper and forearm, shoulder, and back
Kramp [132]	To find an ergonomic difference between two operation setups in a crossover study.	removing a gallbladder in the French and American position (20.8 min)	4 surgeons	3 electromagnetic motion-tracking sensors	head and trunk
Wang [133]	To compare the ergonomics of surgeons using either baseline equipment or an exoscope	autologous breast reconstruction using deep inferior epigastric perforator (DIEP) flap surgery (552.1 min skin-to-skin and 635.5 min exoscope)	2 surgeons	4 IMU	head and upper back

**Table A3:** All the studies on the industrial sector included but with a lower population.

First Author	Aim	Task	Subjects	Instrumentation	Body Segments
Vignais [64]	To analyse material handling task.	Clean, build, vacuum, pack a filter (20 min)	5 workers	7 IMUs and 2 electrogoniometers	head, trunk, upper and forearms, hands
Colim [65]	To present a musculoskeletal risk assessment before and after robotic implementation in an assembly workstation	normal performance of assembly work cycles	11 IMUs	2 workers	head, trunk, upper and forearms, hands
Kersten [126]	To collect information about when, and for how long, specific assembly tasks were performed during up to 14 consecutive work shifts	machine-paced manufacturing (71.8 to 93.7 min)	6 workers	radio frequency identification (RFID) system and 1 IMU	dominant upper arm
Brents [134]	To asses trunk postures using an inertial measurement unit (IMU)-based kinematic measurement system while workers lifted kegs at a craft brewery.	keg lifting	5 workers	17 IMU	Total body
